# Exosomes isolated from IMMUNEPOTENT CRP, a hemoderivative, to accelerate diabetic wound healing

**DOI:** 10.3389/fbioe.2024.1356028

**Published:** 2024-05-20

**Authors:** Paola Leonor García Coronado, Moisés Armides Franco Molina, Diana Ginette Zárate Triviño, Sara Paola Hernández Martínez, Beatriz Elena Castro Valenzuela, Pablo Zapata Benavides, Cristina Rodríguez Padilla

**Affiliations:** ^1^ Laboratorio de Inmunología y Virología, Facultad de Ciencias Biológicas, Universidad Autónoma de Nuevo León, San Nicolás de los Garza, Mexico; ^2^ Facultad de Agronomía, Universidad Autónoma de Nuevo León, General Escobedo, Mexico

**Keywords:** diabetic-wound healing, IMMUNEPOTENT CRP, Akt pathway, collagen expression, exosomes

## Abstract

The increasing risk of amputation due to diabetic foot ulcer calls for new therapeutic options; for that, we determined the role of IMMUNEPOTENT CRP (ICRP) and its parts in the wound healing process of superficial wounds in diabetic BALB/c mice. A potency test was performed to confirm the batch of ICRP, and then its parts were separated into pellets, supernatants, and exosomes, and another group of exosomes loaded with insulin was added. Viability and scratch healing were assessed in NIH-3T3, HUVEC, and HACAT cell lines. Diabetes was induced with streptozotocin, and wounds were made by dissecting the back skin. Treatments were topically applied, and closure was monitored; inflammatory cytokines in sera were also evaluated by flow cytometry, and histological analysis was performed by Masson’s staining and immunohistochemistry for p-AKT, p-FOXO, p-P21, and p-TSC2. ICRP pellets and exosomes increased cellular viability, and exosomes and exosome–insulin accelerated scratch healing *in vitro*. Exosome–insulin releases insulin constantly over time *in vitro*. *In vivo*, treatments accelerated wound closure, and better performance was observed in pellet, exosome, and exosome–insulin treatments. Best collagen expression was induced by ICRP. P-AKT and p-FOXO were overexpressed in healing tissues. Inflammatory cytokines were downregulated by all treatments. In conclusion, IMMUNEPOTENT CRP components, especially exosomes, and the process of encapsulation of exosome–insulin accelerate diabetic wound healing and enhance cellular proliferation, collagen production, and inflammation modulation through the phosphorylation of components of the AKT pathway.

## 1 Introduction

Approximately 529 million individuals worldwide were living with diabetes mellitus (DM) in 2021, and it has been predicted that more than 1.31 billion people will have DM in 2050 (Ong et al., 2023). Among the array of complications stemming from this condition, diabetic foot ulcers (DFUs) stand out as a prominent concern. Every 30 seconds, a limb is amputated due to complications from DM (Jere et al., 2022). In DM, failures at diverse points of the wound-healing process can be observed, inducing DFUs characterized by an inflammatory environment (Falanga, 2004). The process of wound healing consists of four overlapping stages: a) Hemostasis: this initial phase focuses on stopping bleeding through platelet activation, forming a temporary fibrin clot that also serves as a scaffold for subsequent healing processes. b) Inflammation: occurring concurrently with hemostasis, this phase aims to prevent infection. Neutrophils, lymphocytes, and monocytes migrate to the wound site to remove debris and bacteria while secreting growth factors that initiate the next phase. c) Proliferation: taking place around 2–10 days post-injury, this phase involves cell proliferation, angiogenesis, collagen deposition, and wound contraction. Various growth factors and enzymes facilitate these processes, culminating in the formation of granulation tissue. d) Remodeling: beginning approximately 2–3 weeks post-injury and lasting up to 2 years, this phase focuses on scar tissue maturation. Type III collagen is replaced by type I collagen, which enhances tissue strength, though it never fully matches the pre-injury strength (Nosrati et al., 2023).

Conventional treatments include the topical application of growth factors, metformin, and insulin, with the help of non-adherent bandages with collagen and hyaluronic acid (Lee et al., 2016; Park et al., 2019), with insulin being the most effective of the treatments mentioned. Alternatives of new treatments are being explored, among which are exosomes; exosomes are extracellular vesicles of 50–200 nm considered nano-vectors capable of exerting functions similar to the cell of origin (Li et al., 2020). Exosome therapies include mesenchymal/adipose stem cell exosomes in diabetic wounds [ (Zhang et al., 2018; Wang et al., 2021; Li et al., 2020)] that exert biological activity by activating the PI3K-AKT pathway, even more coming from hypoxic adipose stem cells (Wang et al., 2021). The serine/threonine kinase Akt drives diverse cellular functions such as cell cycle regulation, apoptosis control, angiogenesis facilitation, and glucose metabolism. In addition, the PI3K/AKT pathway contributes to glucose metabolism and the wound-healing processes associated with DM (Wang et al., 2022).

IMMUNEPOTENT CRP (ICRP) is a mixture of substances (<12 kDa) from a dialyzate of a bovine spleen leukocyte extract with immunomodulatory capacity. This dialyzate induces antioxidant and anti-inflammatory capacity in human macrophages stimulated with lipopolysaccharides and exhibits a cytotoxic effect in cancer cells *in vitro*; this last property is commonly used as a test of potency for extracts (Franco-Molina et al., 2011; Franco-Molina et al., 2021). In a clinical trial of third-molar extraction, ICRP action was compared with that of ibuprofen, demonstrating that ICRP exerts an anti-inflammatory effect (Franco-Molina et al., 2016). Our research team has *in silico* determined some of the varying biological effects of IMMUNEPOTENT CRP derived from both their overall peptide content and the peptides present within exosomes that promote wound healing. This finding suggests the need for new preclinical investigations aimed at unraveling the activity of IMMUNEPOTENT CRP in the wound healing process by modulating inflammation and activating signaling pathways, including but not limited to PIP3/AKT (García Coronado et al., 2023). For this, we propose to determine whether the wound-healing activity in a diabetic mouse model is dependent on exosomes contained in the IMMUNEPOTENT CRP, from some of the ICRP parts separated through centrifugation, or the total compound. Furthermore, we aim to evaluate the efficiency of insulin encapsulated in exosomes isolated from IMMUNEPOTENT CRP.

## 2 Materials and methods

### 2.1 Exosome isolation and characterization

IMMUNEPOTENT CRP exosomes were isolated by the ultracentrifugation method employing the kit ExoQuick (Invitrogen) and centrifuged at 10,000 X g for 1 h at 4°C. Shape, size, and protein content were reported in a previous study by our research team (García Coronado et al., 2023).

### 2.2 Insulin encapsulation and insulin release rate evaluation

For encapsulation, 100 µL (422.98 µg) of exosomes were mixed with 100 µL (450 μg, insulin) of commercial insulin (AMSA, México) and incubated for 30 min at 37°C. Then, electroporation was performed with a disposable electroporation cuvette at 250 V and 125 µF (in sucrose buffer 600 mM) in an electroporator (BioRad MicroPulser). After that, the cell membrane recovery period was 45 min at 37°C. Subsequently, exosomes were recovered. The pellet (loaded exosomes) was resuspended in 1 mL of PBS 1X and then incubated in lysis buffer (Triton 1X, 1:1 v/v) for 45 min and centrifuged at 10,000 X g for 10 min. The supernatant was collected, while the pellet was resuspended in 1 mL of PBS. The pellet and supernatant were placed in a new 96-well plate to read the absorbance at 280 nm. Previously, a calibration curve was established with insulin (not loaded), and the percentage of encapsulation was calculated using the following formula (Hernández Martínez et al., 2019):

$$Encapsulation\;\left(\%\right)=\frac{\left(total\;insulin\right)-\;\left(free\;insulin\right)}{\left(total\;insulin\right)\;}\;x\;100.$$ (1)

To determine insulin release, loaded exosomes were incubated in a 96-well plate with buffer pH 2 and pH 8 (in triplicate, 1:1 per pH) at different incubation periods (0, 10, 30, 60, and 90 min). Afterward, the absorbance was read at 280 nm, and the percentage of release was calculated with the following formula (Hernández Martínez et al., 2019):

$$\text{Release }\left(\%\right)=\left(\text{free}\;\text{insulin}\right)\;/\;\left(\text{total}\;\text{insulin}\right)\;X\;100.$$ (2)

The results are presented as the mean value of triplicates.

### 2.3 ICRP potency test

The mammary murine cancer cell line 4T1 (ATCC) was employed as a model to corroborate the identity of ICRP in a potency test. 4T1 cells were seeded in a 96-well plate (5 × 10^3^ cells per well) and incubated for 24 h at 37°C and 5% CO_2_. Then, the following treatments were applied: ICRP at different concentrations (1 U, 0.75 U, 0.5 U, and 0.25 U diluted in DMEM 1% antibiotic and anti-mycotic), ICRP pellets (from now on named pellet treatment), and supernatants (from now on named supernatant treatment), both obtained by ICRP centrifugation at 10,000 X g for 60 min at 4°C from 1 U, 0.75 U, 0.5 U, and 0.25 U diluted in DMEM, and the exosomes (isolated from 5 U, 0.75 U, 0.5 U, and 0.25 U diluted in DMEM). Viability was assessed by the resazurin metabolism method (10% (v/v) resazurin); after an incubation period of 1 h, the plate was read at 530 nm excitation/590 nm emission wavelengths.

Subsequently, a recovery viability assay was performed for 24 h with the same treatments and concentrations mentioned above, withdrawing the medium with treatment after this period and replacing it with a new DMEM, evaluating the viability by the resazurin metabolism method at 5 days post-treatment.

As part of the potency test, 4T1 cells (3 × 10^5^/well) were seeded on coverslips located one per well in 6-well plates and incubated for 24 h Then, cells were treated with the ICRP, pellet, supernatant, and exosome treatments at a concentration of 0.5 U/mL in DMEM for 24 h. After that, apoptosis was assessed through staining with acridine orange and ethidium bromide in a 1:1 ratio per well with an incubation period of 30 s. After that, the cells were visualized under a Zeiss fluorescence confocal microscope at 10X. Images were processed using ImageJ software to merge and interpret them by the appearance of colors in a qualitative manner. The green solid nucleus indicated viable cells, the fragmented green nucleus indicated early apoptosis, orange cells indicated late apoptosis, and red indicated necrotic cells. Five photographs were randomly taken from different fields per treatment, and one representative photo was chosen per group. The death process was identified by the presence of each color as a qualitative test. All experiments were performed in triplicate. A number of cells indicating viable cells, early apoptosis, late apoptosis, and necrosis were graphed as a percentage per field.

### 2.4 Conditioned medium

Human peripheral venous blood obtained from a healthy donor was incubated in tubes with 3 mL of blood (heparinized) with ICRP at 0.5 U, 0.25 U, and 0 U for 24 h at 37°C and 5% CO_2_. After this period, blood was centrifuged at 3,500 rpm for 30 min, and the serum was separated. DMEM was supplemented with previously obtained sera at 10% to treat HUVEC cells (5 × 10^3^ cells/well) in a 96-well for 24 h, and the viability was evaluated by the resazurin method in triplicated wells per dose. The experiment was performed in triplicate.

### 2.5 IMMUNEPOTENT CRP *in silico* biological functions prediction

A list of proteins was built (composed list), including a list of commonly expressed proteins in human fibroblasts downloaded from the Human Protein Atlas database and a list of ICRP proteins (used in a previous study). Then, on STRING: functional protein association network online software, the composed list was submitted in the option multiple proteins selecting as organism *Bos taurus*. The MCL infiltration parameter was employed by entering the number 2. The criterion that was used to highlight the biological functions from the interactome hits were those related to improving the healing process, with a strength value greater than 0.5 (Bozhilova et al., 2019).

### 2.6 Viability test

HUVEC, NIH-3T3, and HACAT cells were seeded in 96-well plates at (5 × 10^3^ cells/well) and incubated for 24 h at 37°C and 5% CO_2_. Then, cells were treated with the following treatments: ICRP (1U, 0.75 U, 0.5 U, and 0.25 U diluted in DMEM 1% antibiotic and anti-mycotic), pellet (from 1 U, 0.75 U, 0.5 U, and 0.25 U diluted in DMEM 1% antibiotic and anti-mycotic), supernatant (from 1 U, 0.75 U, 0.5 U, and 0.25 U diluted in DMEM medium 1% antibiotic and anti-mycotic), exosomes (from 1 U, 0.75 U, 0.5 U, and 0.25 U diluted in DMEM 1% antibiotic and anti-mycotic), and insulin as positive control (100%,75%, 50%, and 25% diluted in DMEM 1% antibiotic and anti-mycotic). Then, cells were incubated for 24 h at 37°C and 5% CO_2_. Viability was evaluated using the previously mentioned resazurin metabolism method. Experimental values are presented as the mean value of triplicates.

### 2.7 *In vitro* scratch healing assay

HUVEC, NIH-3T3, and HACAT cells were seeded in 24-well plates (5 × 10^4^ cells/well diluted in the DMEM) and incubated for 24 h at 37°C and 5% CO_2_ until reaching 80% of confluence, and then cell proliferation was synchronized by incubating cells for 5 h with DMEM without fetal bovine serum (FBS). The cell monolayer was then scratched with a sterile spike in the center of the well to simulate a wound. The following treatments were added: ICRP, pellet, supernatant, exosomes, and exosomes loaded with insulin (from now on exosome-insulin), all at a concentration of 5 U in DMEM without FBS. Subsequently, the scratch area was monitored with photographs at 0, 24, and 48 h using ImageJ software, and the scratch healing percentage was calculated with the following formula (Hernández Martínez et al., 2019):

$$scratch\;healing\;\left(\%\right)=\left(A_{0}-\;A_{t}\right)÷\left(A_{0}\;x\;100\right),$$ (3)

where *A0* is the wound area at time 0 and *At* is the wound area corresponding to each time point.

Experimental values are presented as the mean value of five repetitions.

### 2.8 Induction of diabetes in BALB/c mice

Six-week-old BALB/c female mice, (22–26 gr), were kept at a constant temperature of 28°C in a vivarium with *ad libitum* access to food. Diabetes was induced with the protocol of three (daily) doses of streptozotocin intraperitoneally (65 mg/kg of body weight dissolved in citrate buffer pH 4.5) after a 4-h starvation period. Two weeks after the last injection with STZ, a glucose tolerance curve was performed (12 h post fasting and a subsequent dose of oral dextrose, 2 mg/kg of body weight). Blood glucose levels were quantified by using a glucometer (Accu-Chek Instant) every 30 min for 2 h by caudal vein puncture. Mice with glucose levels >160 mg/dL were considered diabetic. All the experimental protocols were submitted for evaluation by the ethics and animal welfare committee of the College of Biological Sciences of the Autonomous University of Nuevo León and were carried out in accordance with the official Mexican standard NOM-062-ZOO-1999, for the production, care, and use of experimental animals.

### 2.9 Wound closure in mice

Mice were anesthetized with an intramuscular injection of anesthesia (ketamine 80 mg/kg; xylazine 5 mg/kg). Afterward, the back was depilated and disinfected with 70% ethanol. Subsequently, a dorsal wound was made by extracting a fragment of skin (0.5 × 0.5 cm). The following topical treatments were diluted in injectable water and applied to the wound site: ICRP, pellet, exosomes, exosome-insulin, insulin (100 µL as positive control), and 1X PBS (as negative control); one daily dose for 5 days. Animals were clinically inspected daily for 21 days, and wound closure was documented by photographs. The area of the wound was measured with a Vernier (measuring height and width), and wound closure was calculated using the following formula:

$$Wound\;closure\;\left(\%\right)=\left(A_{0}-\;A_{t}\right)÷\left(A_{0}\;x\;100\right).$$ ([16c])

### 2.10 Assessment of collagen synthesis

Mice were anesthetized as previously mentioned and sacrificed by cervical dislocation on days 7, 4, and 21. Dermal tissues from the wound area were surgically recovered and fixed in a 10% formaldehyde solution for 24 h and stored in paraffin blocks. Later, they were cut into 5-μm sections and placed on slides to be deparaffinized and rehydrated. To observe the presence of collagen fibers, Masson’s trichrome staining was performed. Photographs were taken at 10X with a Zeiss confocal microscope coupled to an Axiocam camera. Images were processed in ImageJ software with the FIJI plugin ((v. 1.54f, http://imagej.org) to determine the expression percentage of each marker; the expression values were graphed in Prism 9V. Three photographs were randomly taken from different fields per treatment, and one representative photo was chosen per group to build the figure.

### 2.11 Epithelial thickness, epithelial area, and cell number calculation

From treated mice, at days 0, 7, 14, and 21 post-treatment, skin tissue was obtained from the scar site, and hematoxylin and eosin staining was performed. Cells were counted by counting nuclei. This was performed in ImageJ plugin FIJI, separating purple color (corresponding to the hematoxylin signal) with color deconvolution function, and then the binary mask image was generated employing watershed function to separate nuclei that were closely located. Then, nuclei were counted. Nuclei at the edges were excluded from the quantification. The number of cells represents the mean value from three photographs (at random fields) located from the epidermis to the subcutaneous tissue at ×40 objective (Zeiss confocal microscope coupled to an Axiocam camera). Values were graphed in Prism 9V.

Photographs used for the collagen expression analysis (Masson’s trichrome staining) were used to calculate the epithelial thickness and area. The images were then analyzed using ImageJ Fiji software (v. 1.54f, http://imagej.org). For discrete measurements of the epithelial thickness, each image’s file was opened using the software, and proper scales were established according to the previous magnification (2000 pixels = 1 μm). The thickness of each epithelium was measured by selecting the “Straight” option and sketching a line going through the epithelium. This was done so with five random sections of the epithelium for each sample analyzed (three photographs per treatment). Data were retrieved after selecting the “Measurement” command in the “Analyze” tab, and it was further analyzed using GraphPad Prism (v. 9.0.0.).

For measuring the total area of each epithelium, each image’s file was opened using Fiji software, and proper scales were established according to the previous magnification. Once opened and scaled, the “Polygon selections” option was enabled, and the area comprising the whole epithelium for each sample was analyzed and then carefully encompassed within the selection. Data were retrieved after selecting the “Measurement” command in the “Analyze” tab, and data were further analyzed using GraphPad Prism (v. 9.0.0.).

The granulation tissue was analyzed by thickness means. For that, the granulation tissue was identified, then the “Straight” option was selected, and sketched a line going through the thickness was sketched. This was done at three random sections for each sample analyzed. Data were retrieved after selecting the “Measurement” command in the “Analyze” tab, and it was further analyzed using GraphPad Prism (v. 9.0.0.). Scoring of the skin tissue at the wound site post-treatment was established according to the criteria of Greenhalgh et al. (1990) (Supplementary Material S1).

### 2.12 Pro-inflammatory cytokines

Blood was obtained by cardiac puncture of mice (at days 7, 14, and 21), and serum was separated to determine the expression of inflammatory cytokines using the BD Cytometric Bead Array (CBA) Mouse Inflammation Kit (Cat. 552364) following the manufacturer’s instructions. Values were analyzed in the FlowJo program to later graph the data in Prism 9. The experiment was performed in triplicate.

### 2.13 PI3K-AKT pathway activation

The samples from day 7 were evaluated to assess the expression of phosphorylated components of the PI3K-AKT pathway using the VECTASTAIN Elite ABC HRP kit (Vector Laboratories, PK-6100) according to the manufacturer’s instructions. We used the following primary antibodies from Thermo Fisher: AKT-P (PA5-39725), FOXO-P (PA5-118528), P21-P (PA5-99373), and TSC2-P (PA5-104916), and then the presence of markers was revealed through brown coloration produced by the metabolism of the DAB substrate by the peroxidase enzyme (DAB substrate kit, peroxidase (HRP) Vector Laboratories). Photographs were taken on a Zeiss confocal microscope coupled to an Axiocam at 40X and processed in ImageJ software with the Fiji plugin (v. 1.54f, http://imagej.org) to determine the expression percentage of each marker. Five photographs were randomly taken from different fields per treatment, and one representative photo was chosen per group to build the figure. Expression values were graphed in Prism 9V.

### 2.14 Statistical analysis

Statistical analyses were performed using GraphPad Prism version 9 software and data are expressed as the mean ± standard deviation. Statistical significance was determined using a one-way ANOVA test, followed by a Tukey multiple comparison test *post hoc* or T and Wilcoxon test (as applicable) with a significant difference denoted by *p* < 0.05.

## 3 Results

### 3.1 Exosomes isolated from IMMUNEPOTENT CRP do not affect cell viability but induce late apoptosis in 4T1 cells in a potency test

**FIGURE 1:**
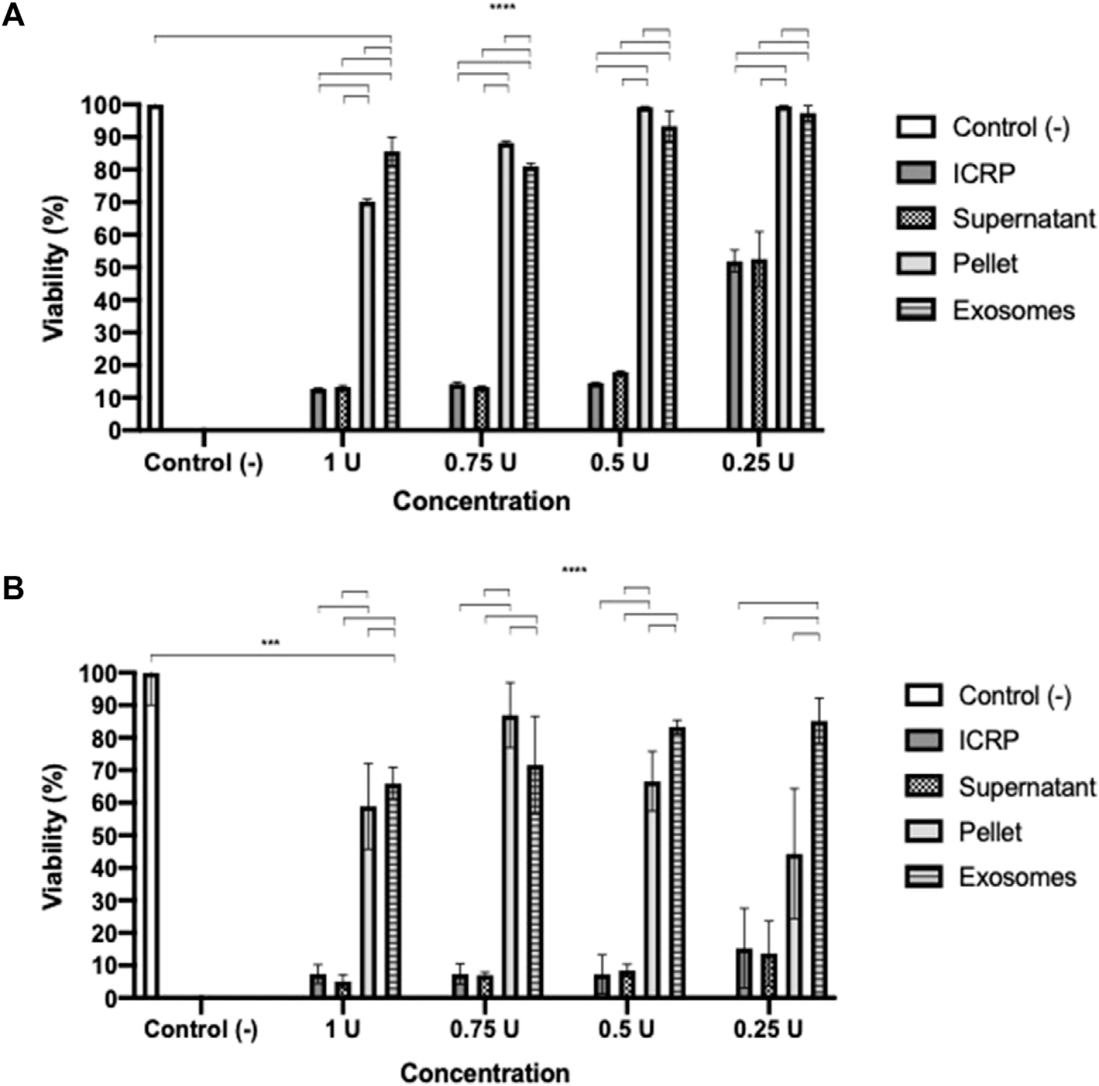
Cytotoxic effect of the ICRP in the 4T1 cell line. **(A).** After 24 h of treatment, cell viability was measured by resazurin metabolism. **(B).** After 24 h of treatment, this was removed and replaced with DMEM; 5 days later, viability was measured by resazurin metabolism. Exosomes do not possess cytotoxic effects in 4T1 cells. Tukey test (*p* < 0.05).

A potency test was performed to assess the effect that each of the ICPR parts has on the viability of the 4T1 murine breast cancer cell line. After 24 h (Figure 1A), the ICRP and ICRP supernatant decreased the viability by 12.76% and 13.26% (*p* < 0.05) respectively, and also induced a cytotoxic effect in a recovery assay (Figure 1B), whereas the exosome and ICRP pellet did not significantly affect the cell viability (70.94% and 99.5%, respectively).

A poor cytotoxic effect was reflected at 24 h of treatment with both exosomes and pellets, in a recovery assay (Figure 1B), and the cytotoxicity increased in a dose- and treatment-dependent manner (*p* < 0.05). Exosomes (from 1U) showed a cytotoxicity of 34.1% and pellet of 41.08%. The ICRP cytotoxic effect in the 4T1 cell line is due to the mixture of all its parts.

**FIGURE 2:**
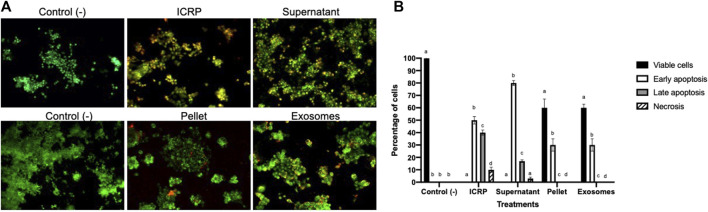
Exosomes isolated from the ICRP promote late apoptosis. **(A)**. Dyeing with acridine orange and ethidium bromide was performed in 4T1 cells after being treated with 0.5 U derived treatments for 24 h. The green solid nucleus indicates viable cells, the fragmented green nucleus indicates early apoptosis, orange cells indicate late apoptosis, and red indicates necrotic cells. Representative photos, 10X Zeiss fluorescence microscope. **(B)**. Percentage of cells belonging to each cell death process, number of cells was calculated as a percentage per view field, and letter represent significant differences within groups Tukey test (*p* < 0.05).

The findings were corroborated by acridine orange and ethidium bromide staining to determine ICRP-induced apoptosis (Figure 2). Similar effects were observed with the supernatant, whereas in exosome and pellet treatment, early apoptosis was observed 24 h post-treatment at a dose that induced a poor cytotoxic effect in the recuperation test.

### 3.2 IMMUNEPOTENT CRP increases cell viability in the HUVEC cell line

**FIGURE 3:**
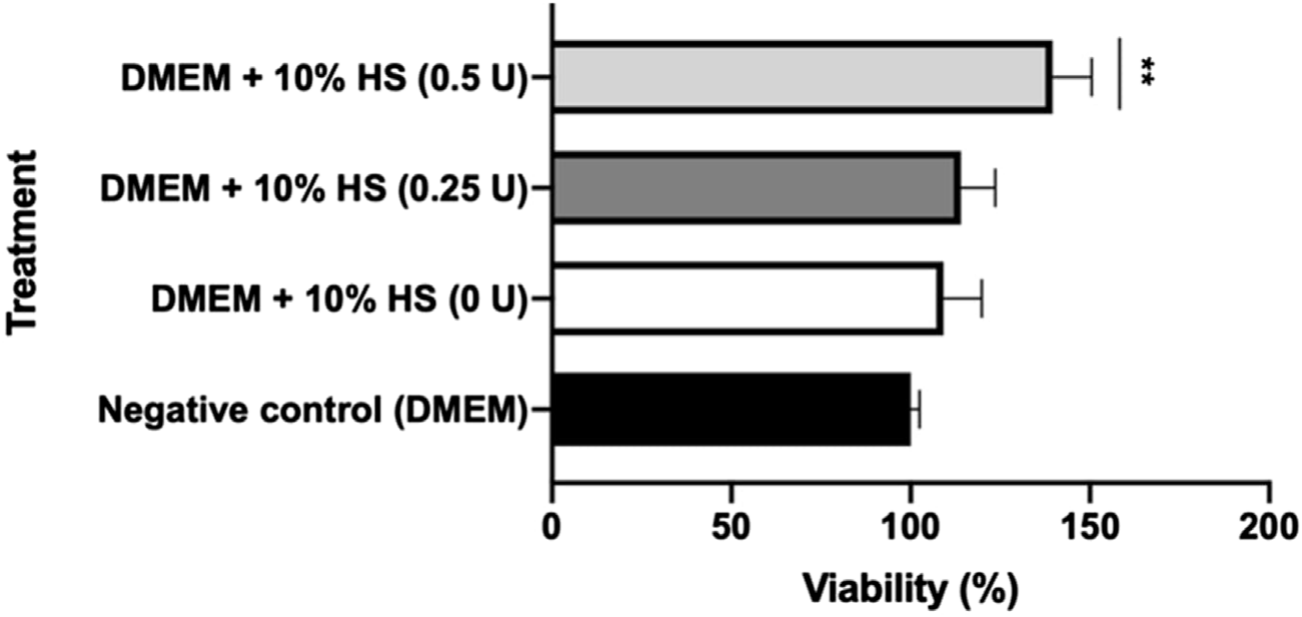
Conditioned medium from blood treated with ICRP promotes HUVEC cell line proliferation. Peripheral blood was treated with ICRP (0 U, 0.25 U, and 0.5 U) for 24 h; after that, serum was separated and became supplement for DMEM (at 10%) to cultivate HUVEC cells. Viability was measured after 24 h. HS. Conditioned medium. *t*-test and Wilcoxon test were performed (*p* < 0.001) (n = 3).

The ICRP is a complex mixture of substances, with exosomes included within the extract. The question arises as to whether ICRP as a treatment on peripheral whole blood cells could induce the release of factors and molecules that could promote cell proliferation and be involved in the wound healing process. The conditional medium induced proliferation in a dose-dependent manner at 24 h, post-treatment (Figure 3) with a significant difference in the negative control. The conditional medium derived from treated blood with ICRP 0.5 U induced viability up to 140% (Figure 3), and ICRP 0.25 U treatment did not show a statistical difference against the control (*p* < 0.05). Another plausible fact is that cells cultured with DMEM with no sera did not show a difference against cells cultured with DMEM containing 10% human sera derived from blood not treated with ICRP (*p* < 0.05).

### 3.3 Whole IMMUNEPOTENT CRP participates in wound healing processes, and its exosomes increase cell viability in dermal cells

**FIGURE 4:**
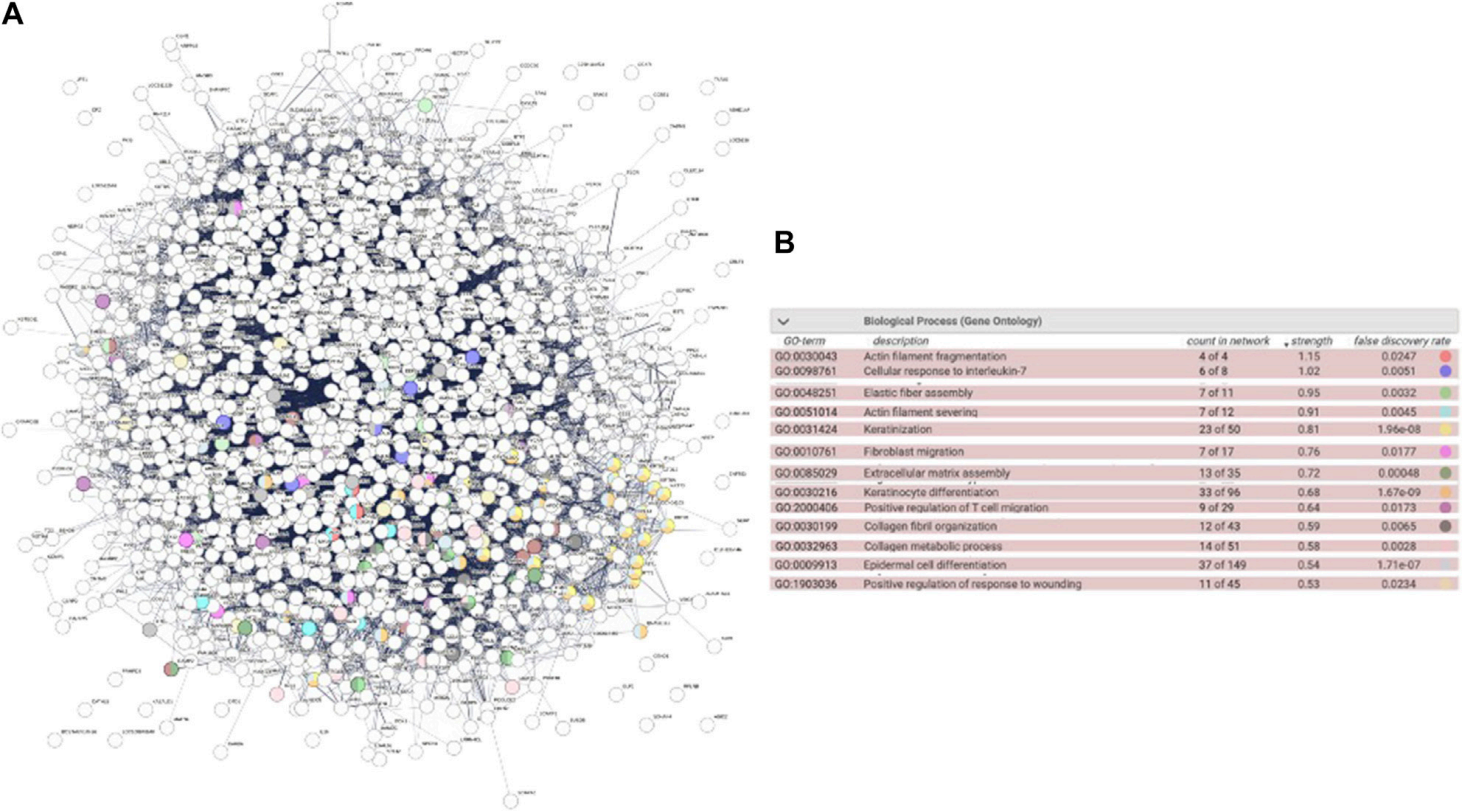
Biological functions induced by proteins in the ICRP in fibroblast cells. Biological functions highlighted showed a strength value greater than 0.5 in STRING functional protein association networks. **(A)**. Interactome. **(B)**. Highlighted biological functions related to wound healing.

**FIGURE 5:**
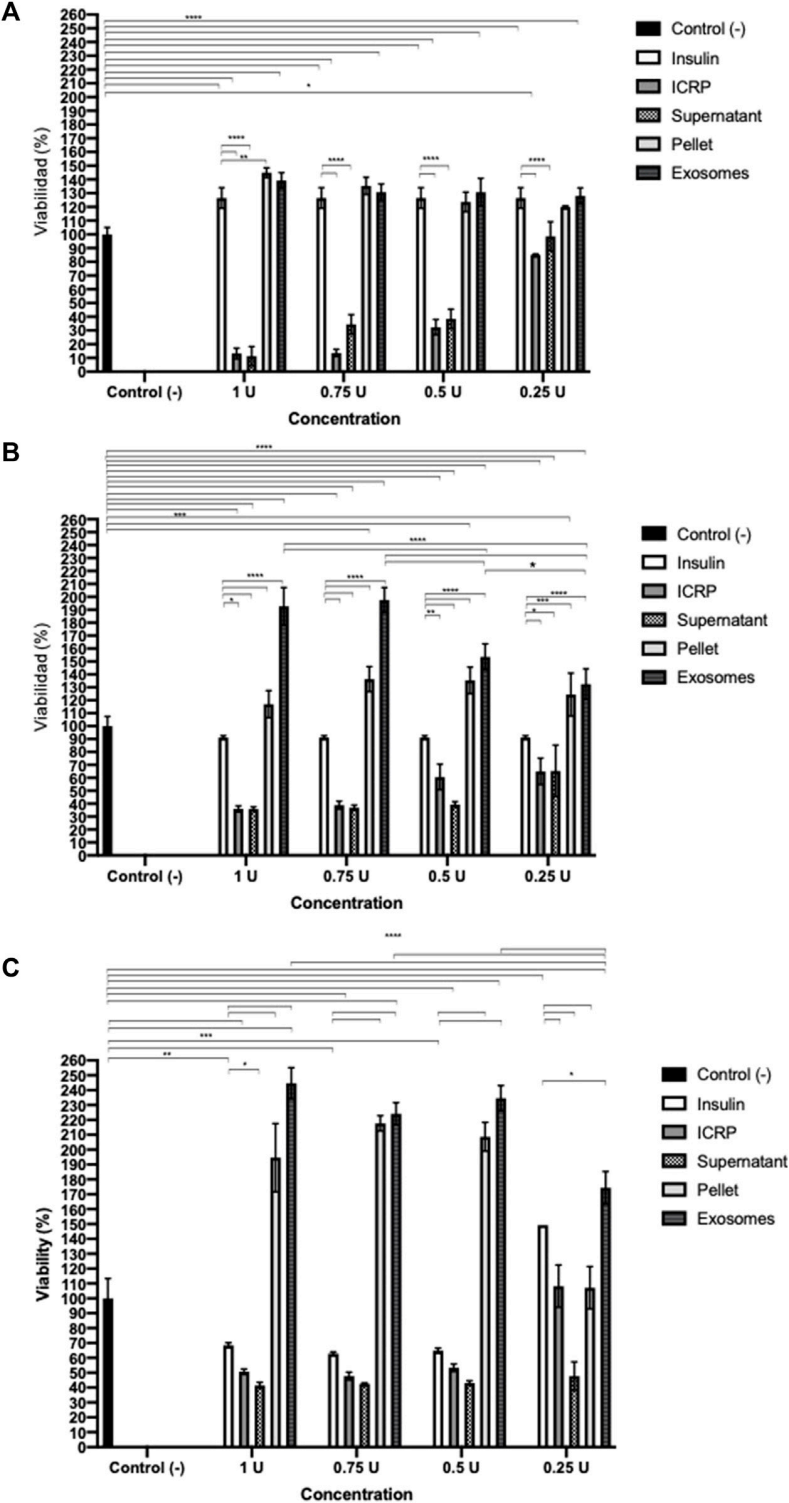
Effect of the ICRP’s exosomes increases cell viability. 5 × 10^3^ cells were seeded and treated, after 24 h, viability was measured by resazurin metabolism. **(A)**. Exosome treatment isolated from 1 U of ICRP promotes proliferation up to 139% on HUVEC cell lines. **(B)**. Exosome treatment isolated from 1 U of ICRP promotes proliferation up to 193% on the NIH-3T3 cell line. **(C)**. Exosome treatment isolated from 1 U of ICRP promotes proliferation up to 244% on HACAT cell line. Tukey test (*p* < 0.05) (n = 3).

The ICRP as a whole extract has a cytotoxic effect on cancer cell lines, but conditioned media induce proliferation in the HUVEC cell line. The next questions were: was this effect attributed to a part of the extract or the product as a whole? Could the parts in the ICRP (one of them, exosomes) induce proliferation in cell lines that form part of the dermis layers, for future wound healing process purposes? *In silico* predictions indicated the activation of biological functions related to wound healing with high confidence based on a strength value greater than 0.5 in STRING functional protein association networks for fibroblast cells (Figure 4), highlighting actin filament fragmentation, cellular response to IL-7, elastic fiber assembly, actin filament severing, and keratinization (Figure 4B). For that, the viability post-treatment was measured in HUVEC, NIH-3T3, and HACAT cell lines. It could be observed in Figure 4 that exosome treatment increased viability in all cell lines in a dose-dependent manner, with a statistical difference against the control (*p* < 0.05). HUVEC cells treated with pellets and exosomes showed an increase in the viability (144% and 139%, respectively) with significant differences against the negative control at all concentration treatments, but not against the positive control, that is, insulin (*p* < 0.05) (Figure 5A). ICRP treatment diminished viability with statistical (*p* < 0.05) difference against exosomes. NIH-3T3 cells (Figure 5B) treated with exosomes increased the viability up to 193% in the 1 U dose with statistical differences against positive and negative control. Additionally, differences (*p* < 0.05) between treatments could be observed at all doses. In addition, for the HACAT cell line, the highest viability percentage was observed in exosome treatment with a value of 244% 24 h after treatment (1 U doses, Figure 5C), with statistical differences (*p* < 0.05) observed against negative and positive control, in a dose-dependent manner. Another notable treatment in the induction of proliferation is pellet treatment, which in the HUVEC cells, viability was induced up to 144.9% at 1 U dose of treatment, with statistical differences (*p* < 0.05) against positive and negative control. In the NIH-3T3 cell line, 136.5% viability was induced at 0.75 U with a significant difference against controls. In the HACAT cell line, 271.69% viability was induced at 0.75 U, with a significant difference against controls. At median doses induce a better proliferation effect (*p* < 0.05) (Figure 5). A tendency is observed in three cell lines, and the two best treatments are pellet and exosomes.

The highest cytotoxic effect in 4T1 cells was observed in supernatant treatment from 1 U in the recovery assay, with 5.02% of viable cells. Dermal cells (HUVEC, NIH-3T3, and HACAT cell lines) treated with 1 U of different derived treatments showed viability, but the one with the highest values was exosome treatment, except for pellet treatment in the HUVEC cell line. ICRP that contains exosomes induced higher proliferation in the last three mentioned cell lines than the positive control, insulin (Table 1).

**TABLE 1:** Viability post-treatment with ICRP and its components (%).

Treatment (24 h post-treatment)	1 U				0.75 U				0.5 U				0.25 U				Insulin
	1	2	3	4	1	2	3	4	1	2	3	4	1	2	3	4	NA
4T1	12.79	13.26	70.24	85.66	14.17	13.31	88.23	81.00	14.54	17.89	99.2	93.35	51.91	52.56	99.5	97.35	NA
4T1 recovery assay	7.36	5.02	58.92	65.91	7.32	7.05	86.94	71.59	7.27	8.42	66.59	83.31	15.27	13.64	44.34	85.14	NA
NIH-3T3	35.95	35.90	117.06	192.68	38.89	36.97	136.35	197.55	60.71	39.43	135.40	153.62	65.02	65.35	124.43	132.56	91.19
HUVEC	13.23	11.25	144.92	139.24	13.69	34.46	135.32	130.77	32.45	38.51	123.61	130.81	85.11	98.54	120.05	128.17	126.49
HACAT	50.76	41.53	194.61	244.61	47.69	42.56	217.69	224.10	53.33	43.07	208.71	234.61	108.20	47.69	107.17	174.35	149.23

### 3.4 Exosomes isolated from IMMUNEPOTENT CRP can encapsulate insulin

**FIGURE 6:**
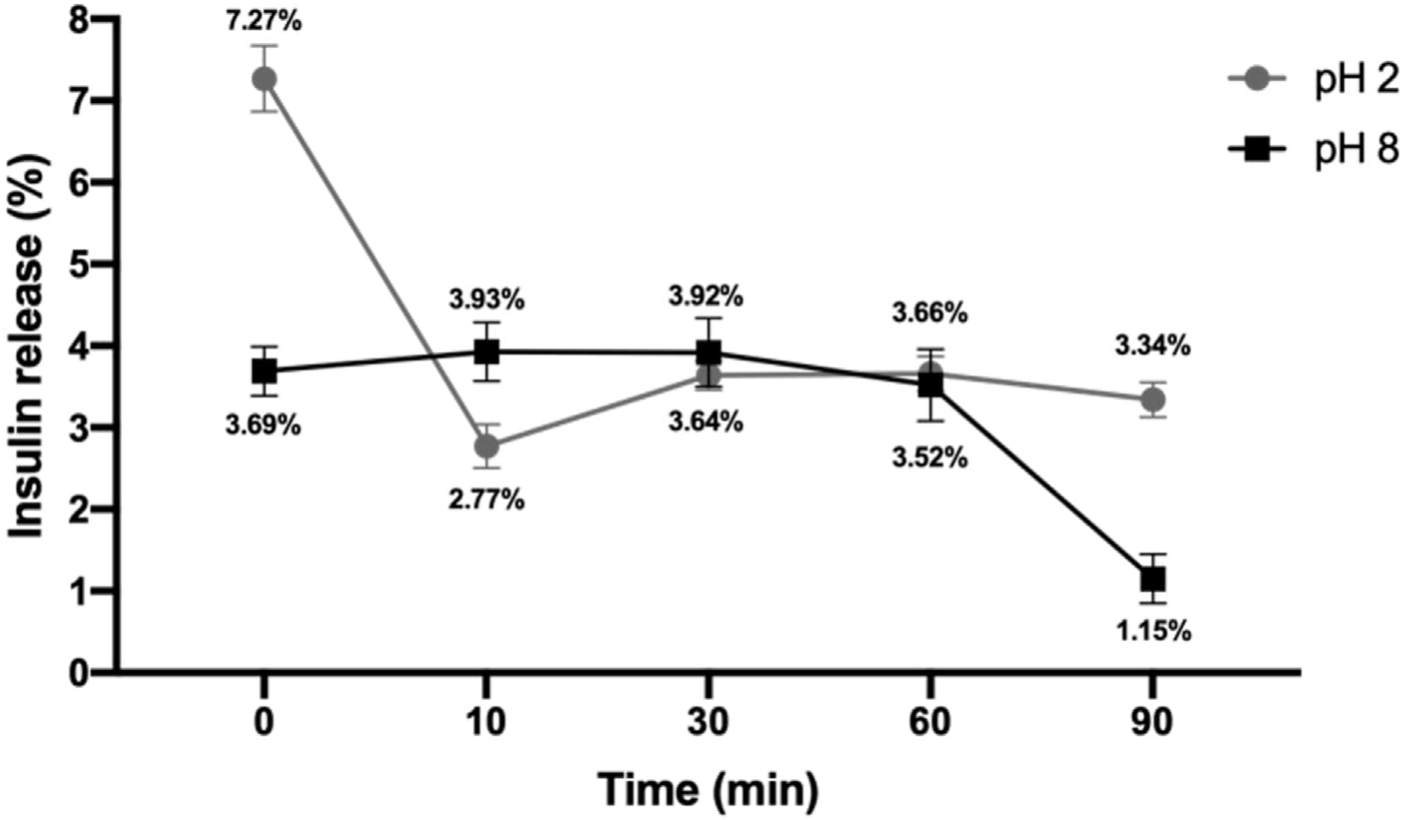
Insulin release percentage. Loaded exosomes were subjected to buffer with acid pH (pH 2) or basic pH (pH 8), and insulin release was monitored over time by measuring the absorbance at 280 nm (n = 3).

Exosomes are considered nano-vectors that can carry substances; for this reason, insulin was loaded through electroporation. The exosome’s capacity to load insulin and the ability to constantly release it was evaluated, and an encapsulation efficiency of 86.08% was achieved (Table 2). Mostly, exosomes released insulin at pH 8 with similar values at pH 2, both with a stable constant rate of release over time (Figure 6). Only at pH 2 a peak in release was observed, equivalent to a 7.27% in minute 0. From now on, loaded exosomes are named exosome-insulin.

**TABLE 2:** Insulin encapsulation in exosomes isolated from the ICRP negative control was considered 100% of viability, numbers indicate the treatment derived from 1 U, 0.75 U, 0.5 U, and 0.25 U of ICRP, considering insulin as the positive control. 1. ICRP, 2. supernatant, 3. pellet, and 4. exosomes.

Equation of the line	y = 415.12x - 1123.3
Supernatant absorbance at 280 nm	3.0635
Substitution of the equation of the line	148.42012
Percentage of encapsulation	86.08%

### 3.5 Exosomes isolated from IMMUNEPOTENT CRP promote cell proliferation and migration in dermal cells

**FIGURE 7:**
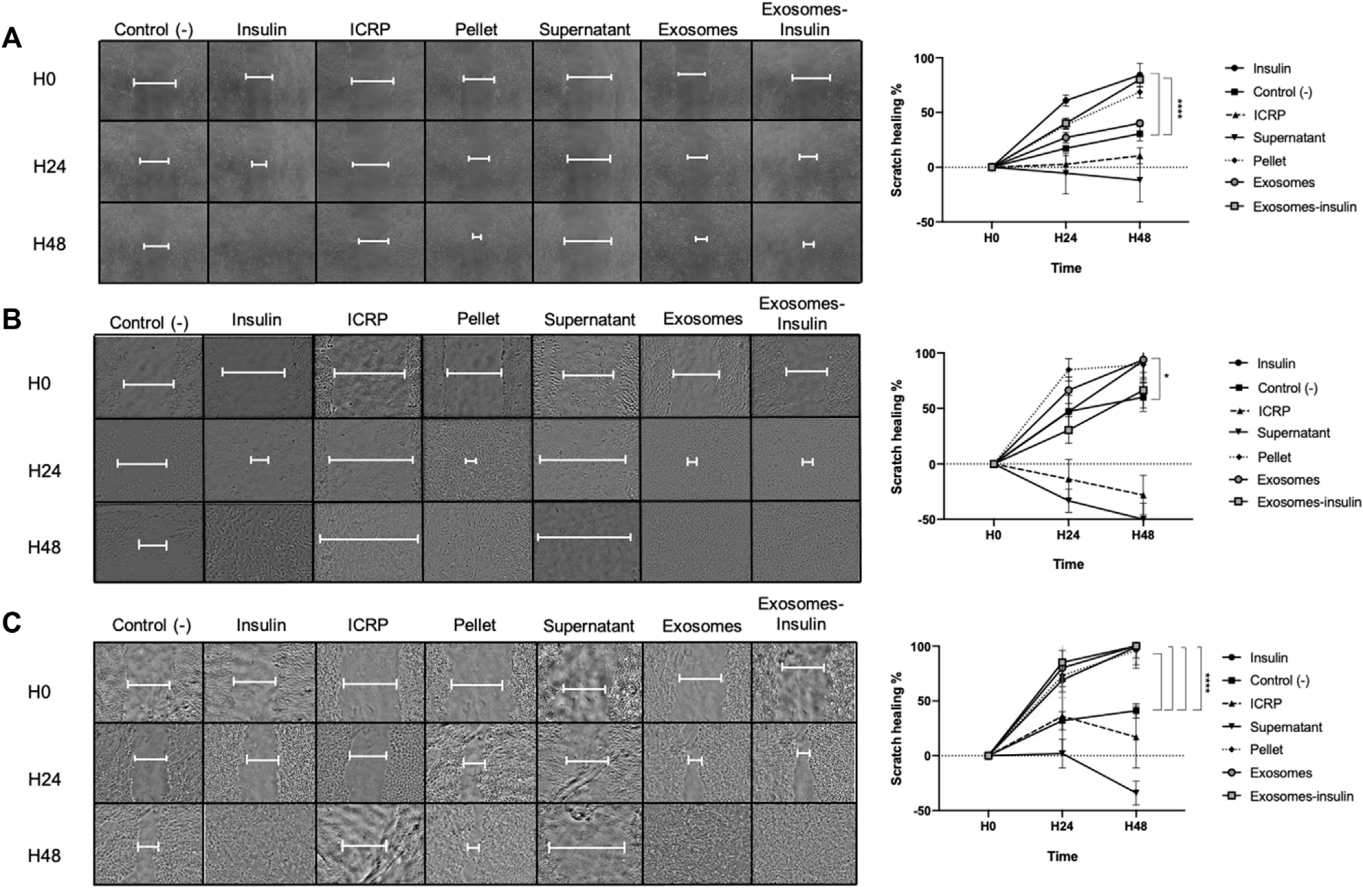
Exosomes alone and exosome-insulin stimulate cell proliferation and migration. Scratch was made in the cellular monolayer, and then treatments were applied; the scratch area was monitored through photographs taken at 0, 24, and 48 h **(A)**. Exosomes and exosomes induce scratch healing in the HUVEC cell line. **(B)**. Exosomes and exosomes induce scratch healing in the NIH-3T3 cell line. **(C)**. Exosomes and exosome-insulin induce scratch healing in the HACAT cell line. Photographs were analyzed in ImageJ, and statistical analysis was performed with Tukey’s test (*p* < 0.05) (n = 5).

The same cell lines were treated with previous treatments plus one that was exosome-insulin to assess if one part of the ICRP was able to accelerate the closure of a scratch in a monolayer. ICRP and the supernatant increased the scratch area in three cell lines, whereas exosomes and exosome-insulin treatments did not show statistical differences against positive control (insulin) accelerating the closure of the scratch. In the HUVEC cell line, both insulin and exosome-insulin treatments healed the scratch fastest with a statistical difference (*p* < 0.05) against negative control at 48 h (Figure 7A). In the NIH-3T3 cell line, exosome-insulin treatment did not show a better performance than exosomes or insulin (as best treatments) as in HUVEC cell line, which showed significant differences against control (Figure 7B). Pellet treatment in all cell lines also was a good inductor of proliferation and migration, similar to exosomes and exosome-insulin at 24 h. There was no significant difference (*p* < 0.05) between exosomes and exosome-insulin compared with insulin (positive control). At this stage, based on conditioned medium, viability, and scratch assay, the best treatments proposed to be used in the *in vivo* phase were ICRP, pellet, exosomes, and exosome-insulin.

### 3.6 Exosomes isolated from IMMUNEPOTENT CRP charged with insulin promote wound healing in an *in vivo* model

**FIGURE 8:**
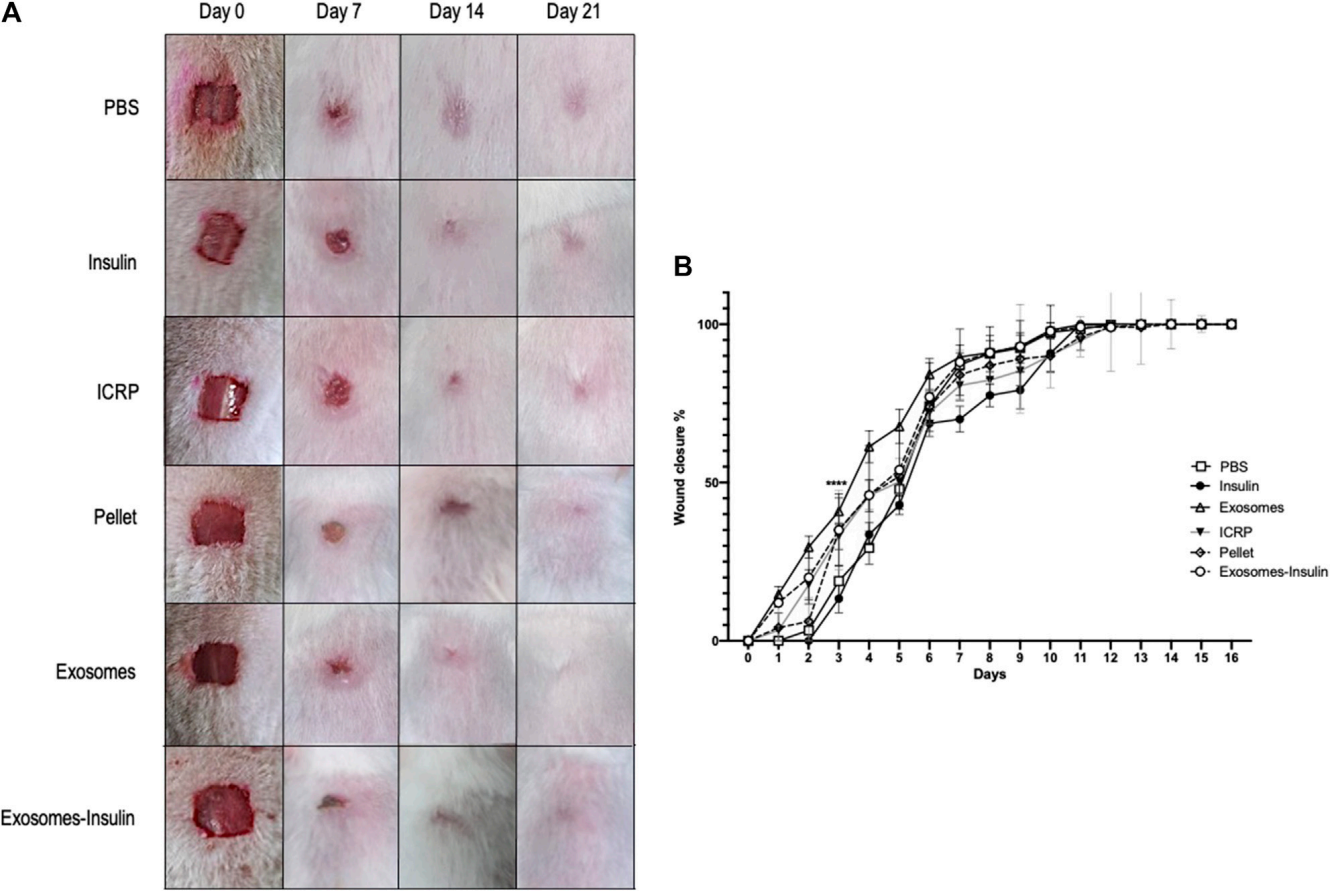
Exosomes and exosome-insulin treatment promote an accelerated wound closure. **(A)**. Representative photos of mice during the wound healing process after five doses of their respective treatment. **(B)**. Exosomes and exosome-insulin treatments accelerate wound closure, and statistical differences between all treatments were observed at day 3. The wound healed at day 12. Tukey Test (*p* < 0.05) (n = 5).

In *in vivo* probes, it could be observed that mice treated with exosomes exhibited the fastest healing with increased hair growth on the skin surrounding the wound (Figure 8A), compared to negative control (PBS) or positive control (insulin). In pictures, it could be observed that the skin appears closed but with pink coloration, indicating the closure of the wound but not wound healing, due to the contraction of the panniculus carnosus muscle. Other outstanding treatments were ICRP and pellet by day 4, followed by exosome-insulin treatment (the second-best treatment), implying modulation of wound closure during the first days. All wounds healed by day 12, but differences between treatments were found on the first 5 days; at day 3, all treatments had statistical differences, with the exosome and exosome-insulin treatments showing a higher percentage of wound closure, 67% and 54%, respectively. In addition, ICRP treatment and pellet treatment showed 50% and 52% of wound closure, respectively, with a good performance, compared to PBS and insulin (47% and 42%) at the same day; over time, differences between treatments decreased, but a tendency was observed in which exosomes showed the best performance (Figure 8B); in the remaining days, no significant difference was observed between exosomes and exosome-insulin treatments.

**FIGURE 9:**
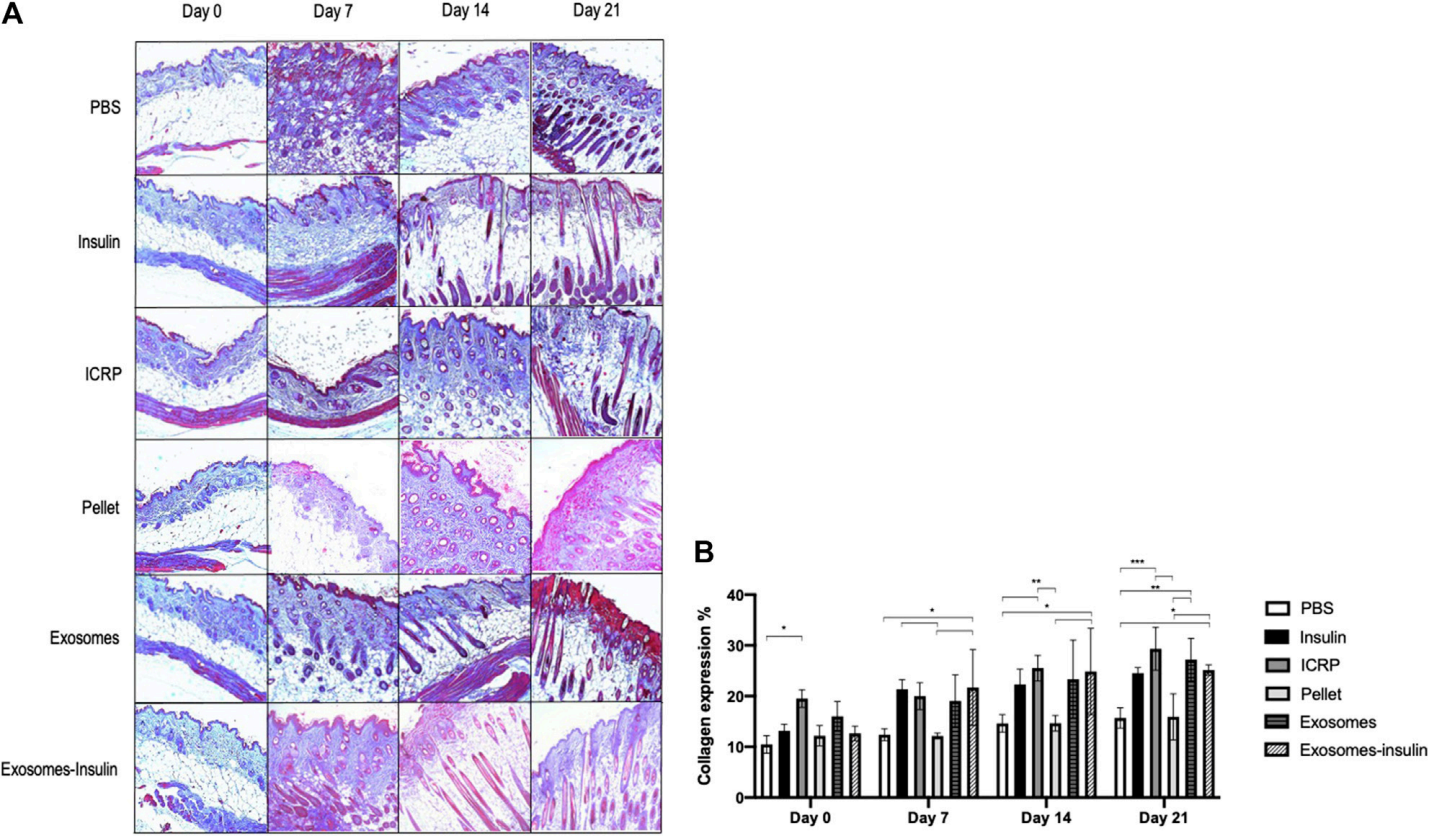
Exosomes and exosome-insulin treatments induce an accelerated wound healing. **(A)**. Masson’s trichrome staining was performed on histological sections of mice subjected to the different treatments. Photos taken at 10X. Red: muscle/erythrocytes, blue: collagen, pink: fibrin, blue/black: nuclei. **(B)**. Collagen expression quantified at ImageJ, Tukey test (*p* < 0.05) (n = 3).

The exosome and exosome-insulin treatments induced an accelerated wound healing process, but the question remains if underneath this layer, dermal layers were organized as another sign of proper wound healing. For that, trichrome staining was performed, and the blue coloration indicated that collagen expression was observed on all days in all treatments (Figure 9A). At day 21, the higher expression of collagen fibers was observed, with the highest production in mice treated with ICRP (29.32%), followed by exosome and exosome-insulin treatments with 27.19% and 25.12%, respectively, with significant differences against the negative control (Figure 9B). Over time, an increase in collagen could be observed in all treatments, except for pellet treatment: at day 0, 12.17% of expression; 12.13% at day 7; 14.64% at day 14; and 15.90% at day 21. Dermal layers of mice treated with ICRP, exosomes, and exosome-insulin seemed more organized concerning the negative control and comparable to the positive control (Figure 9A), especially in mice treated with exosomes and exosome-insulin, driving a more esthetic appearance (Figure 9A).

On the other hand, at day 7, more fibrin (pink) was observed in all treatments, indicating the inflammation phase; the pellet treatment exhibited the thinnest pink layer at the top of the skin, indicating that mice treated with pellets modulated the best inflammation at day 7. The presence of capillary follicles as an indicator of closure could be observed, especially in mice treated with exosomes and exosome-insulin at day 7. By day 14, hair follicles could be observed in mice treated with exosomes, exosome-insulin, and insulin. The first ones presented larger cells surrounding hair follicles (Figure 9A).

**FIGURE 10:**
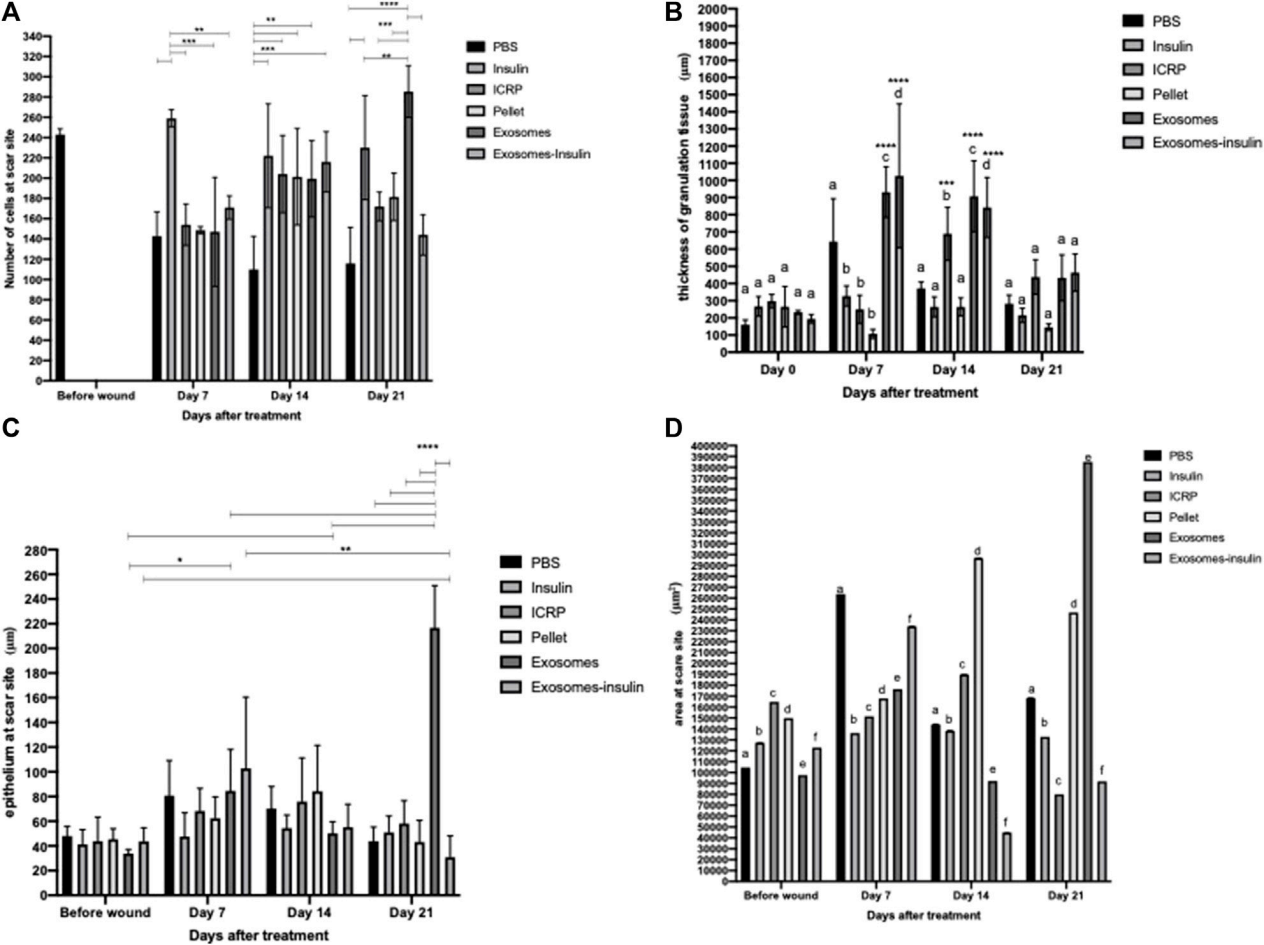
Induction of healing by ICRP and its components. **(A)**. Number of cells at the wound site, the number of nuclei was analyzed in ImageJ, asterisks show significant differences within the group (Tukey test *p* < 0.05 n = 3). **(B)**. Thickness of the granulation tissue in μm was analyzed using the polygonal line in ImageJ, the letters show significant differences within groups, and the asterisks differences between groups (Tukey test *p* < 0.05, 5 measures per photograph, n = 3). **(C)**. Thickness of the epithelium at the wound site in μm was analyzed using the polygonal line in ImageJ, and the asterisks show significant differences within groups and between groups (Tukey test *p* < 0.05, five measures per photograph n = 3). **(D)**. Area of the epithelium at the wound site in μm^2^, the epithelium was outlined and its area was measured in ImageJ, and letters represent statistical differences within and between groups (Tukey test *p* < 0.05, n = 3).

It was observed that the number of cells at the wound/scar site increased depending on the treatment and the day (Figure 10A). Significant differences were observed within groups on days 7, 14, and 21 (Figure 10A). On day 7, mice treated with insulin (positive control) had a greater number of cells compared to those with other treatments (Figure 10A). On day 14, the mice treated with PBS had the lowest number of cells compared to the other treatments (Figure 10A). On day 21, mice treated with insulin or exosomes had a greater number of cells compared to the negative control, and at the same time, mice treated with exosomes had a greater number of cells than the positive control (Figure 10A). No differences were observed between groups: the treatments did not increase or decrease the number of cells compared to the skin tissue without wound (Figure 10A) (*p* < 0.05).

An indicator of the healing phase is granulation tissue for the inflammation and proliferation phase. Therefore, the thickness of the granulation tissue was measured. It was observed that on day 0, there were no differences within the group, while on days 7 and 14, there were differences within the group (Figure 10B) (*p* < 0.05). On day 7, granulation tissue with larger thickness is observed in mice treated with PBS, exosomes, and exosome-insulin (Figure 10B). On day 14, there is no difference between the mice treated with PBS, insulin, and pellet, but there is an increase in the thickness of the granular tissue in the mice treated with ICRP, exosomes, and exosome-insulin (Figure 10B) (*p* < 0.05). At day 21, there were no differences in the thickness of the granulation tissue (Figure 10B). Differences were even observed between groups compared to those on day 0 (Figure 10B) (*p* < 0.05). On day 7, it was observed that the mice treated with exosomes or exosome-insulin show an increase in the thickness of the granular tissue compared to day 0, while on day 14, the mice treated with ICRP, exosomes, or exosome-insulin also showed an increase in the thickness of the granular tissue compared to day 0, with no differences compared to day 7 (Figure 10B) (*p* < 0.05).

It is observed that during the first days, there were no differences in the epithelium thickness between the groups, until day 21, where the groups of treated mice did present differences compared to the negative control group (PBS), indicating a greater thickness of the epithelium, indicative of a scar in remodeling, presenting a greater thickness in the treatment of exosomes. It was also observed that the exosome treatment increased the epithelium thickness, with significant differences on days 7 and 14 in the wound compared to day 0 (Figure 10C). Regarding the area of the epithelium, significant differences are observed within and between groups (*p* < 0.05). On day 7, the treatment that presented the greatest thickness in the epithelium was PBS, following the exosome-insulin treatment (Figure 10D). On day 14, the treatments that presented epithelium with the smallest thickness were exosomes and exosome-insulin, with the pellet treatment providing the largest thickness (Figure 10D). On day 21, the treatments that presented epithelium with the largest thickness were pellets and exosomes (Figure 10D). According to the criteria of healing (Table 3), the mice treated with exosomes or exosome-insulin showed better healing process, with epithelium regeneration.

**TABLE 3:** Scoring of skin tissue at the wound site post-treatment (Hernández Martínez et al., 2019) (Supplementary Material S2).

Treatment	Score day 7	Score day 14	Score day 21
PBS	3	4	7
Insulin	4	8	9
ICRP	5	7	8
Pellet	4	6	7
Exosomes	7	10	11
Exosome-insulin	7	10	11

### 3.7 Topical treatment with IMMUNEPOTENT CRP exosomes modulates cytokine expression in diabetic mice

**FIGURE 11:**
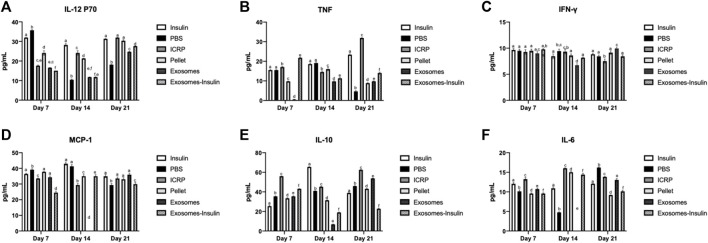
Inflammatory cytokine expression in diabetic mice treated topically at the wound site. Blood was obtained on days 7, 14, and 21 and sera separated and evaluated through flow cytometry. Tukey test (*p* < 0.05) (n = 3). **(A)** IL-12 p70. **(B)**. TNF. **(C)**. IFN-γ. **(D)**. MCP-1. **(E)**. IL-10. **(F)**. IL-6.

ICRP can modulate the immune system: ICRP and its parts could modulate the cytokine expression systemically after being topically applied at the wound site. Expression of IL-12 P70 after 5 days of treatment decreased at days 7 and 14; by day 21, all treatments increased the IL-12 P70 expression, except for insulin treatment, which remained above 28.23 pg/mL on all days (Figure 11A). Treatments resulting in lower IL-12 P70 expression were PBS with 10.46 pg/mL, exosomes, and exosome-insulin with 11.79 pg/mL; statistical differences were observed between all treatments, except in exosomes and exosome-insulin at day 14 (Figure 11A). The pro-inflammatory cytokine TNF significantly increased in mice treated with ICRP, with the highest concentration of 31.86 pg/mL at day 21 with a tendency to increase with time. In contrast, mice treated with exosomes showed statistically the lowest expression of TNF (0.49 pg/mL) (Figure 11B) at day 14. Regarding the IFN-γ expression, at day 7, no significant differences against control were observed between treatments at day 7 (Figure 11C). On day 14, the mice treated with exosomes showed lower expression, while ICRP treatment showed the lowest expression at day 21 (6.73 pg/mL) (Figure 11C).

The chemokine MCP-1 expression in sera was downregulated by the exosome-insulin treatment at day 7 (24.52 pg/mL), after the ICRP treatment (33.56 pg/mL) concerning PBS treatment (39.18 pg/mL) (Figure 11D). At day 14, no expression was observed in mice treated with exosomes, but was observed in mice treated with ICRP (29.37 pg/mL) (Figure 11D), with statistical differences against the control. Anti-inflammatory cytokine IL-10 was overexpressed in all treatments at day 7 with statistical differences against controls, the treatment with the highest expression was ICRP with 55.94 pg/mL, followed by exosome-insulin with 43.08 pg/mL (Figure 11E). IL-6 cytokine was expressed in 10.91–12.07 pg/mL (Figure 11F). At day 7, all treatments had statistical differences, and the mice treated with pellets and exosomes-insulin showed lower expression levels at 9.57 pg/mL (Figure 11F). At day 14, PBS treatment decreased the expression to 4.75 pg/mL and no differences in expression in exosome treatment were observed. The ICRP treatment had the highest expression (16.03 pg/mL) (Figure 11F). At day 21, the treatments with lower expression were pellet with 9.19 pg/mL and exosome-insulin with 10.14 pg/mL (Figure 11F).

### 3.8 Exosomes isolated from IMMUNEPOTENT CRP charged with insulin or alone activate the AKT pathway to promote wound healing

**FIGURE 12:**
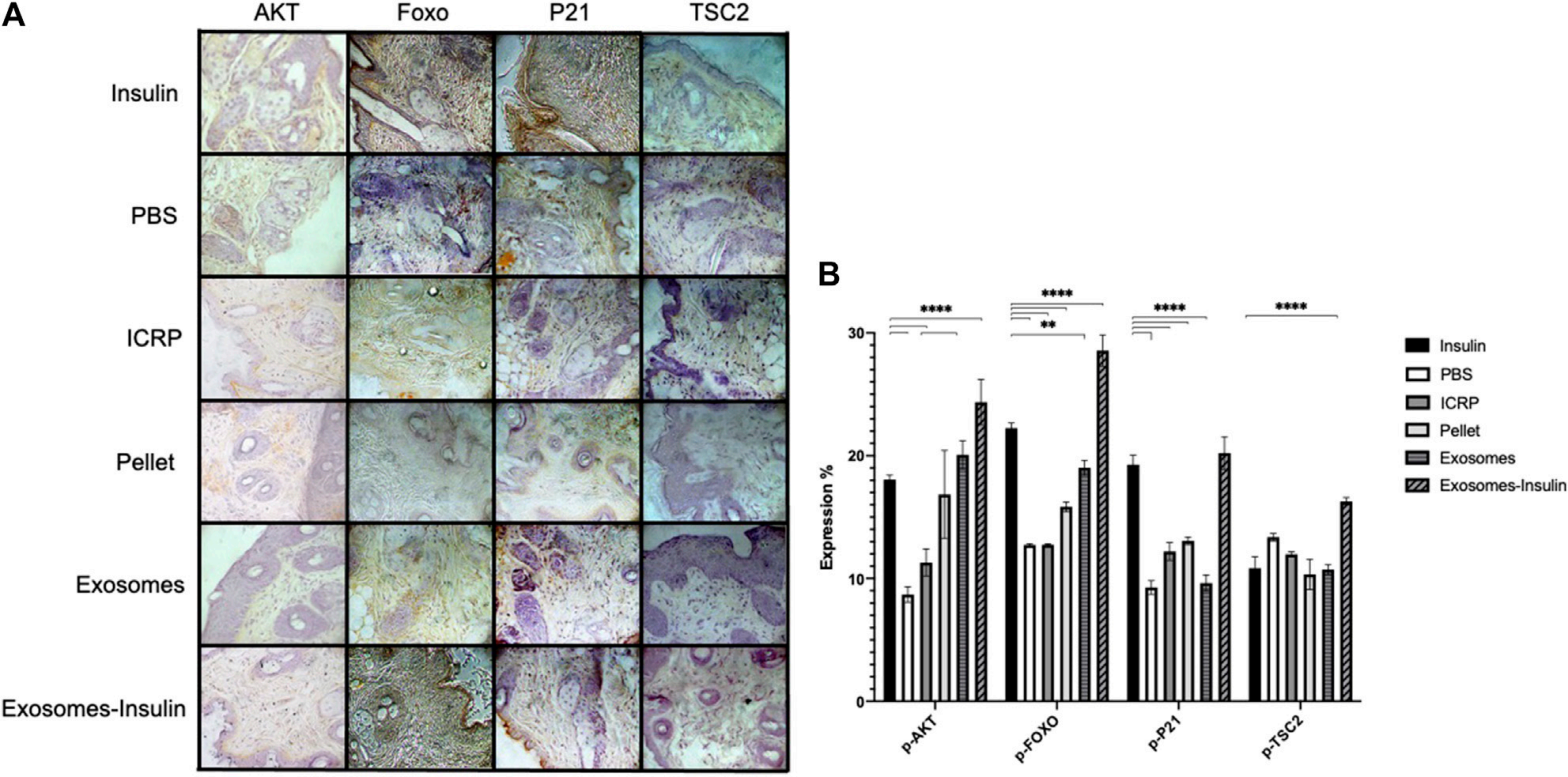
Excisional wounds in diabetic mice treated with exosomes and exosome-insulin heal faster through activation of the AKT pathway. **(A)**. Wound were treated topically for 5 days; at day 7, pieces of skin were cut at the wound site and fixed with formaldehyde 10%, and then immunohistochemistry was performed and contrasted with hematoxylin. Representative photographs at 10X taken with a Zeiss optical microscope attached to a camera are shown. **(B)**. Photographs were analyzed in the ImageJ program to calculate the expression percentage of each marker; mean values were presented in the graph. Tukey test (*p* < 0.05) (n = 5).

Diabetic mice treated topically with exosome-insulin showed increased expression of p-AKT with 24.36% compared to insulin with 18.05% with statistical differences (*p* < 0.05) (Figure 12B). The negative control showed the lowest expression with 8.7%. ICRP containing exosomes showed an expression of 11.22%, whilst exosomes showed 20.07% (Figure 12B). The p-FOXO expression checks the activation of the pathway: negative control and ICRP showed the same expression (12%), whereas insulin showed an expression of 22.24%, while exosome-insulin treatment demonstrated the highest expression of 28.55% with statistical differences against insulin (*p* < 0.05) (Figure 12B). Mice treated with PBS and exosomes showed a low expression of p-P21 of 9%, whereas mice treated with insulin showed an expression of 19.27%, compared to the highest expression, with statistical differences in exosome-insulin treatment with 20.21% (*p* < 0.05) (Figure 12B); ICRP and pellet also showed statistical differences against insulin, with 12.2% and 13.07% (Figure 12B). The other negative marker of pathway activation p-TSC2 appeared lower than negative control PBS (below 13.36%), except for exosome-insulin treatment with 16.28%, being the only treatment with statistical difference (*p* < 0.05) against insulin treatment with 10.83% (Figure 12B). Immunohistochemical analysis indicated activation of the PI3-AKT pathway (Figure 12A).

### 3.9 Discussion

This study discusses diabetic wound healing and the components of the IMMUNEPOTENT CRP. Diabetic foot ulcers present a significant challenge within the healthcare sector due to potential complications, including limb amputation. While therapeutic options exist, they are often insufficient for advanced cases of ulceration. We propose exploring exosomes from IMMUNEPOTENT CRP to accelerate healing and corroborate, in part, previous results that assessed exosome functions *in silico* (García Coronado et al., 2023). Prior to this study, it remained unclear if the cytotoxic effects of IMMUNEPOTENT CRP were attributed to its exosomes or its different components obtained by centrifugation (supernatant or pellet) because the cytotoxic test on 4T1 breast cancer cell lines revealed that exosomes are not the main bioactive in charge of the cytotoxic effect on cancer cells.

We confirmed the validity of our batch to be used in our study because IMMUNEPOTENT CRP had a cytotoxic effect on the 4T1 cell line, as previously reported both *in vitro* and *in vivo* (Santana-Krímskaya et al., 2020; Reyes-Ruiz et al., 2021), observing this effect both in the viability test at 24 h and recovery assay 5 days post-treatment; cytotoxicity was observed (87.21%) when treating 5 × 10^3^ 4T1 cells with 1 U of ICRP, correlating with Reyes-Ruiz et al. (2021), observing 86% of cytotoxicity when treating 4T1 cells with ICRP. Therefore, it was verified through the power test that we were working with the registered product. Some interesting effects were also observed when separating ICRP components. When testing the supernatant fraction, a greater cytotoxic effect is observed in the concentration of 1 U of treatment than in the ICRP as a complete extract, as it is well-known that components of an extract when isolated can in some cases increase their functional effect (Juncan et al., 2021). Another component of the ICRP is exosomes. No reports exist about the use of exosomes isolated from a hemoderivative such as a leukocyte extract like the ICRP, but there are reports of exosomes isolated from macrophages, cells abundant in the spleen. Other exosomes have been tested in 4T1 cells, not showing toxic effects but proliferation, except when loaded with paclitaxel, as monitored by the MTT methodology (Wang et al., 2019). On the other hand, exosomes isolated from crab hemolymph were tested on 4T1 cells, monitoring viability through resazurin metabolism, which decreased by 20% after 48 h of treatment with 0.1 mg/mL (Rezakhani et al., 2021). The equivalent similar decrease in viability with our exosomes was observed when administering 542.92 mg/mL (0.75 U), demonstrating that we needed a larger dose to observe the same cytotoxic effect, suggesting the poor cytotoxic effect of our exosomes over 4T1 cells. The effect was verified by the recovery assay 5 days post-treatment by decreasing viability to 70%, a difference of only 10% with the results of 24 h post-treatment. In 4T1 cells treated with 0.5 U of exosomes or pellets, it could be observed through OA and EB staining of fragmented nuclei. Meanwhile, 4T1 cells treated with ICRP and supernatant showed necrosis and apoptosis; a similar effect was previously reported when treating MCF-7 cells with 0.66 U of ICRP, with the majority of cells presenting apoptosis (orange or green with fragmented nuclei cells) but also the presence of necrotic cells (red cells) (Franco-Molina et al., 2006).

Although exosome effects vary according to the cell of origin, their potential role in wound healing is novel, especially from a hemoderivative like IMMUNEPOTENT CRP. Previous investigations evaluated possible functions of exosomes isolated from the IMMUNEPOTENT CRP *in silico* (Franco-Molina et al., 2016) by our research group, proposing a role in biological processes such as wound healing. The study involved both *in vitro* and *in vivo* evaluations, revealing distinct cellular responses to ICRP components. First, we created a conditioned medium for HUVEC cells obtained from human peripheral whole blood previously treated with 0.5 U of ICRP; this induces a greater proliferation in HUVEC cells compared to the control. This technique is based on the molecules that a cell can secrete under treatment, with the potential of inducing proliferation.

Different types of stimuli can promote the activation of biological functions related to survival. It has been reported that stimulating mononuclear cells from cord blood with radiation promotes a pro-angiogenic effect on progenitor endothelial cells *in vitro* (Cervio et al., 2013). Exosomes derived from rat plasma on irradiated macrophages induced angiogenesis (HUVEC cells) by stimulating the immune microenvironment (Li et al., 2021). On the other hand, mesenchymal cells are present in the stroma of various tissues and organs, for example, the spleen and umbilical cord. These cells secrete factors that can be used as a conditioned medium for culture-starved HUVEC cells, inducing proliferation and anti-apoptotic effect. These conditions of stress and hypoxia are present in a diabetic foot ulcer, in which the malformation of vessels (Bader et al., 2014) is a key target to treatment for inducing cellular regeneration. Likewise, platelet-rich plasma has been tested in fibroblast and keratinocytes in an *ex vivo* model, inducing proliferation and accelerating scratch wound closure, due to the presence of growth factors and cytokines (Stessuk et al., 2016; Bakadia et al., 2023). Probably, the exosomes derived from the bovine spleen cells contain these molecules secreted in conditions of hypoxia (as the ICRP comes from a bovine spleen leukocyte extract), as already ICRP had an effect on HUVEC cells in a conditioned medium, which is why it was then tested over fibroblast and keratinocytes directly.

We observed that exosomes potentiated proliferation *in vitro*; thus, we measured their viability indirectly through the metabolism of resazurin, indicating a higher mitochondrial activity, with viability up to a maximum of 244% at 24 h post-treatment. It has been reported that exosomes derived from adipose mesenchymal stem cells are internalized by fibroblasts to increase their proliferation and migration with overexpression of collagen I and III (Hu et al., 2016). This also happens in human dermal fibroblasts (Zhang et al., 2018), correlating with our results in which proliferation was increased in the NIH-3T3 cell line treated with exosomes, with up to 193% viability post-treatment. Exosomes from adipose stem cells have also been reported to induce proliferation in high glucose-induced HUVEC cell lines (Liang et al., 2022); in our experiment, these cells showed 139% of viability 24 h post-treatment. In addition, exosomes derived from adipose stem cells have been reported to accelerate proliferation in the HACAT cell line through highly expressed microRNAs by activating the PI3K/AKT pathway (Yang et al., 2020a); in these cells, our team observed 244% viability 24 h post-treatment. All cell lines compared to controls used closed scratch wounds faster, and statistically same as the insulin used as the positive control; this correlates with different authors using fibroblasts, endothelial vascular cells, and keratinocyte cells (Hu et al., 2016; Yang et al., 2020a; Liang et al., 2022). Moreover, when using exosomes from adipose stem cells undergoing hypoxia, the closure of scratch wounds is faster than with exosomes from adipose stem cells without hypoxia (Wang et al., 2021). IMMUNEPOTENT CRP, due to its manufacturing process, subjects the spleen to hypoxia, so exosomes in the ICRP are the product of hypoxic cells, matching with the results of Wang et al. (2021) who found a similar amount of scratch healing at 24 h in exosomes and exosome-insulin treatment. In addition to that, DFUs are characterized for presenting a hypoxic environment, for that is important to consider a treatment that could work under these conditions for inducing healing. Because insulin was used as a positive control and has a poor half-life in the skin, we decided to encapsulate it in exosomes, obtaining 86.08% of encapsulation and a peak in insulin release (7%) at pH 2, but still stable release at both pHs, proposing that exosomes are a good vector for insulin release to increase its half-life in the skin, ensuring insulin release as DFUs commonly present at an alkaline pH (Yang et al., 2020a). For that, exosome-insulin treatment was used in the consequent experiments.

Another sign of good healing is production of collagen. To test this, we induced hyperglycemia in BALB/c female mice using streptozotocin, as a type 1 diabetes model. Then, we treated topical wounds and observed that exosome treatment caused faster healing, but the highest expression of collagen was by the ICRP treatment at day 21, with 29.32% collagen expression. Second, exosome treatment (27.19%) and tertiary exosome-insulin (25%) caused higher expression than the positive control (24.5%). This correlates with exosomes derived from mesenchymal stem cells as a treatment for wounds in non-diabetic rats, in which exosomes accelerated closure faster than their positive control (MesenGro hMSC medium), and also higher expression of collagen I and III (Zhang et al., 2015). Another interesting fact about exosomes is that when they originate from perivascular cells, they induce higher expression of collagen at the wound site in rat models (Kim et al., 2021), explaining why our exosomes, which originate from an organ, are showing such results. A commonly activated pathway in diabetic wounds treated with exosomes is the PI3/AKT, also reported in a previous *in silico* study of the putative function of exosomes from ICRP (García Coronado et al., 2023). In diabetic mice treated with exosomes and exosome-insulin at the wound site, we observed an activation of PI3/AKT, by the presence of phosphorylated AKT and a consequent phosphorylation of FOXO, whereas in mice treated with ICRP, the expression of mentioned markers was lower than that in the positive control but higher than that in the negative control, suggesting that the active component that induces healing of diabetic wounds from our *in silico* results corresponds to exosomes, confirming our predictions (García Coronado et al., 2023) and correlating with other studies with the same hyperglycemic mice model (Zhang et al., 2018; Wang et al., 2021).

In addition, a consequence of the proliferation PI3/AKT pathway is the modulation of inflammation; we observed that exosomes modulate the inflammatory cytokines in some like the ICRP as a whole extract in human macrophages (Franco-Molina et al., 2005). The IL-12 p70 is secreted by antigen-presenting cells for polarizing macrophages to the M1 phenotype, which is not wanted in wounds. Mice treated with ICRP, exosomes, and exosome-insulin decreased serum expression of IL-12 p70 at days 7 and 14, showing the lowest expression in exosome-insulin, coinciding with faster healing and better organization in dermal layers; this correlates with a study employing IL-12 KO mice that showed accelerated wound healing (Matias et al., 2011). TNF is commonly overexpressed in non-healing wounds (Dhamodharan et al., 2019); it was observed that mice treated with exosomes on day 7 showed decreased levels of TNF and on day 14 mice treated with ICRP. Exosomes and exosome-insulin also showed decreased levels of TNF, this correlates with research in which murine peritoneal macrophages stimulated *in vitro* with LPS when treated with 0.5 U/mL of ICRP also decreased TNF expression (Franco-Molina et al., 2005). These results correlate with a study in which DFUs were treated with hyperbaric oxygen therapy, and only those treated that showed decreased TNF levels healed faster than the control (Semadi, 2019). No statistical differences were observed in the IFN-γ expression at day 7, and it has been reported that IFN-γ expression does not influence the healing process nor the progression of type I diabetes (De George et al., 2023). However, the IFN-γ KO mice demonstrated that defects in IFN-γ impaired wound healing; this could be correlated with decreased IFN-γ expression in mice treated with exosomes (Kanno et al., 2019), being one of the treatments with a better clinical response.

Augmented MCP-1/CCL2 is correlated with accelerated wound healing in diabetic mice models by restoring macrophage response (Wood et al., 2014). Contrary to that, we observed that mice treated with ICRP or exosome-insulin decreased the expression of MCP-1, whereas those mice were part of the treatment with accelerated healing. On the other hand, an observation study indicated that the 2518 A/G genotype is more frequent in human patients with DFU T2DM, with a mutant form of MCP-1 that is highly expressed in serum correlated with low expression of VEGF and retarded wound healing (Li, 2018). The fact that exosomes and exosome-insulin downregulate the expression of MCP-1 is beneficial, due to the probability that patients could express this genotype. It has been proposed that the DFU biomarker develops a risk of low expression of ApoA1 and IL-10 (Nanda et al., 2022). In a mice model of myocardial infarction with IL-10 KO, exosomes derived from endothelial progenitor cells did not exhibit myocardial repair (Yue et al., 2020). In addition, another study on exosomes derived from the same cells reported that IL-10 overexpression promotes regenerative tissue repair in diabetic mice through STAT3 via regulating fibroblast-specific hyaluronan synthesis (Short et al., 2022). This correlates with the increased expression we observed in mice treated with ICRP, exosome-insulin, and exosomes in decreasing order, and also with downregulation of IL-10 in murine peritoneal macrophages stimulated with LPS and then treated with ICRP (Franco-Molina et al., 2005). IL-6 must be considered as it has dual behavior; in our study, it behaved as pro-inflammatory, and one of the characteristics in patients with DM is chronic inflammation. Patients with DFU express high levels of IL-6 (Galicia-Garcia et al., 2020), this being one obstacle in wound healing of DFU as it blocks macrophage polarization. Exosomes derived from autologous dermal fibroblasts have been reported to accelerate diabetic cutaneous wounds through the activation of the AKT-beta catenin pathway, downregulating inflammation by reducing IL-6 expression at wound sites in diabetic rats (Han et al., 2021). In our study, mice treated with pellet and exosome-insulin showed decreased IL-6 expression at day 7, correlating it with accelerated wound healing due to control of the pro-inflammatory environment.

Based on the histological analysis, cell number was maintained during the healing phases, but within groups, differences could be observed, as on day 21 when mice treated with exosomes exhibited a higher number of cells compared to controls; this could imply a higher remodeling of the wounded tissue. The granulation tissue is important as it is one of the components of the proliferation phase (days 4–21); for that, the thickness was measured instead of the number of granulation tissues, as it has been reported to be a better healing parameter (Van de Vyver et al., 2021); it could be observed that at day 7, the mice that exhibited higher thickness of granulation tissue were the ones treated with exosomes and exosome-insulin, at a faster time than that of the rest of treatments, by day 14. ICRP treatment induced a higher thickness of the granulation tissue; at day 21, no difference was observed against day 0, implying that tissue underwent remodeling and maturation phase; it has been reported that exosomes derived from umbilical cord-derived mesenchymal stem cells stimulate skin regeneration by enhancing regeneration of granulation tissue and upregulation of VEGF (Yang et al., 2020b), matching with our results where treated groups presented vascular formation (table 3). In addition, this could be explained that some of the main components of this tissue are endothelial cells and fibroblasts, and the last one was demonstrated to be stimulated by the ICRP components. Another parameter of proper wound healing is the development of the epithelium; we observed that exosomes and exosome-insulin treatment showed a higher thickness in the epithelium, indicating induction of healing, at day 21, higher than that of the tissue before the wound, leading to scar formation; it has been reported that exosomes derived from umbilical cord mesenchymal stem cells can improve the epithelial thickness in ovariectomized rats (Zhang et al., 2023).

The peptides present in the ICRP and/or exosomes modulate the activation of cells of the innate immune system, inferring this as a consequence of the modulation of cytokines. It stimulates cells to release mediators that promote healing processes such as cornification mentioned in previous studies (García Coronado et al., 2023). It has been proven that by using peptides attached to matrices such as hydrogels, the activation of these cells can be enhanced (Griffin et al., 2021). In our study, exosomes were used as nano vectors since, due to their size, they can enter the cells. Cytokine modulation was observed, such as cytokine expression by macrophages. It downregulates IL-12 p70 and IL-6, promoting an M2 phenotype of macrophages, which promote healing by secreting TGF-β, a key cytokine in the regulation of collagen synthesis and other components of the extracellular matrix. PDGF is a cytokine that stimulates fibroblast proliferation and migration (García Coronado et al., 2023). It also upregulates IL-10, which promotes hyaluronic acid synthesis. ICRP peptides and exosomes also directly induce the proliferation of endothelial cells for the formation of blood vessels, a key point in skin nutrition, and induce the same effect in fibroblasts increasing collagen synthesis and providing tensile properties to the skin. It also stimulates keratinocytes, the main component of the outer layers of the skin (cornification and keratinization), providing resistance, impermeability, and protection. These latter results coincide with the *in silico* predictions shown in this research.

In conclusion, the main purpose of this research was to determine which part of the ICRP could induce wound healing or be the main bioactive in charge of that, determining that the IMMUNEPOTENT CRP has a role in wound regeneration; its components as pellet and exosomes, especially last ones, enhance this process in a murine diabetic model. In addition, the exosomes can be used as an efficient nanovector to encapsulate and release insulin to improve healing. The ICRP and/or its parts possess properties such as acceleration of diabetic wound healing, enhanced proliferation, collagen production, and inflammation modulation through the phosphorylation of components of the PI3/AKT pathway. However, more studies are necessary to evaluate the angiogenesis and ulcer tissue perfusion and corroborate these results in diabetic human wounds. One of the limitations of the study is the lack of isolation of the compounds belonging to each of the parts of the ICRP. The perspective remains to isolate them and test whether the peptides determined *in silico* are responsible for the wound healing effect when they are administered. The research’s alignment with previous studies reinforces the findings and suggests avenues for further exploration and potential clinical applications.

## Data Availability

The original contributions presented in the study are included in the article/Supplementary Material; further inquiries can be directed to the corresponding author/s.
