# Supplementary material for: Exosomes isolated from IMMUNEPOTENT CRP, a hemoderivative, to accelerate diabetic wound healing
Source: Front Bioeng Biotechnol. 2024 May 20;12:1356028. doi: 10.3389/fbioe.2024.1356028 (PMC11149424; doi:10.3389/fbioe.2024.1356028)
Supplement: Supplementary file 1 [file DataSheet1.docx]

**Supplementary table 1**. Scoring of histology sections for the skin tissue from wound site stained with Trichrome staining (Greenhalgh *et al*., 1990).

| Score | Criteria |
| --- | --- |
| 1-3 | None to minimal number of cells. No granulation tissue present, nor epithelial travel. |
| 4-6 | Thin / immature granulation tissue dominated by inflammatory cells. Few fibroblasts, capillaries or collagen deposition. Minimal epithelial migration. |
| 7-9 | Moderately / thick granulation tissue. Dominated by inflammatory cells to fibroblast and collagen deposition. Extensive neovascularization. Epithelium from minimal to moderate. |
| 10-12 | Thick vascular granulation tissue, dominated by fibroblasts and extensive collagen deposition. Epithelium tissue partially / completely covering the wound. |


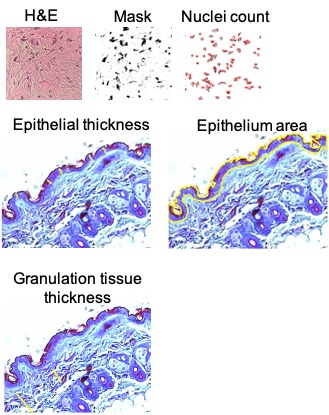


c

b

a

**Supplementary Fig. 1.** Histological analysis for cell count, epithelial thickness, epithelium area, and granulation tissue thickness. **a.** Cell number was determined by nuclei count in FIJI, separating purple color (from H&E staining), creating a binary mask. Mean values from 3 representative photographs per treatment were graphed. **b.** epithelium was analyzed in terms of thickness and area. Epithelial thickness was evaluated using a yellow straight line across the epithelium and length measured in imageJ (5 measurement at different point in the tissue). Epithelium area was measured by contouring the area with polygon section. Measurements performed in 3 representative photographs with trichrome staining per treatment. **c.** Granulation tissue thickness was evaluated using a yellow straight line across and its length measured in imageJ (5 measurement at different point in the tissue). All measurements were performed after scale was set (2000 pixels = 1 µm) in imageJ.
